# Role of Nonalcoholic Fatty Liver Disease in Periodontitis: A Bidirectional Relationship

**DOI:** 10.7759/cureus.63775

**Published:** 2024-07-03

**Authors:** Hardika S Vegda, Bhavin Patel, Gaurav A Girdhar, Mohd. Shabankhan H Pathan, Rahnuma Ahmad, Mainul Haque, Susmita Sinha, Santosh Kumar

**Affiliations:** 1 Department of Periodontology and Implantology, School of Dentistry, Karnavati University, Gandhinagar, IND; 2 Department of Physiology, Medical College for Women and Hospital, Dhaka, BGD; 3 Department of Research, Karnavati Scientific Research Center (KSRC) School of Dentistry, Karnavati University, Gandhinagar, IND; 4 Department of Pharmacology and Therapeutics, National Defence University of Malaysia, Kuala Lumpur, MYS; 5 Department of Physiology, Enam Medical College and Hospital, Dhaka, BGD

**Keywords:** nonalcoholic fatty liver disease, inflammatory bowel disease, low socioeconomic status, oral hygiene status, distorted lipid metabolism, dyslipidemia, insulin resistance, obesity, chronic generalized periodontitis, nafld

## Abstract

Nonalcoholic fatty liver disease (NAFLD) and periodontitis share common risk factors such as obesity, insulin resistance (IR), and dyslipidemia, which contribute to systemic inflammation. It has been suggested that a bidirectional relationship exists between NAFLD and periodontitis, indicating that one condition may exacerbate the other. NAFLD is characterized by excessive fat deposition in the liver and is associated with low-grade chronic inflammation. There are several risk factors for the development of NAFLD, including gender, geriatric community, race, ethnicity, poor sleep quality and sleep deprivation, physical activity, nutritional status, dysbiosis gut microbiota, increased oxidative stress, overweight, obesity, higher body mass index (BMI), IR, type 2 diabetes mellitus (T2DM), metabolic syndrome (MetS), dyslipidemia (hypercholesterolemia), and sarcopenia (decreased skeletal muscle mass). This systemic inflammation can contribute to the progression of periodontitis by impairing immune responses and exacerbating the inflammatory processes in the periodontal tissues.

Furthermore, individuals with NAFLD often exhibit altered lipid metabolism, which may affect oral microbiota composition, leading to dysbiosis and increased susceptibility to periodontal disease. Conversely, periodontitis has been linked to the progression of NAFLD through mechanisms involving systemic inflammation and oxidative stress. Chronic periodontal inflammation can release pro-inflammatory cytokines and bacterial toxins into the bloodstream, contributing to liver inflammation and exacerbating hepatic steatosis. Moreover, periodontitis-induced oxidative stress may promote hepatic lipid accumulation and IR, further aggravating NAFLD. The interplay between NAFLD and periodontitis underscores the importance of comprehensive management strategies targeting both conditions. Lifestyle modifications such as regular exercise, a healthy diet, and proper oral hygiene practices are crucial for preventing and managing these interconnected diseases. Additionally, interdisciplinary collaboration between hepatologists and periodontists is essential for optimizing patient care and improving outcomes in individuals with NAFLD and periodontitis.

## Introduction and background

Periodontitis is an inflammatory chronic tooth disorder initiated by poly-pathogenic microbes remaining in plaque biofilm [1-3]. Periodontitis is also known as grave gum infectious disorder. Chronic periodontitis is influenced by various factors like genetic predisposition, age, alcohol, smoking, obesity, calculus, low socioeconomic status, systemic diseases (e.g., diabetes mellitus, cardiovascular disease, osteoporosis, etc.), and the availability of pathogenic microbes [4-6]. This leads to the destruction of supporting tissues and alveolar bone [7], which, if the patient does not receive appropriate healthcare, can, in turn, lead to tooth loss [8]. It is the sixth most prevalent human disease, which affects approximately 11.2% of the population [9]. The mouth cavity's homeostatic equilibrium is lost because the oral microbiome is disintegrated [10-13]. Pathobionts (opportunistic pathogenic microbes) enter a healthy microbiome, instigating the development of a dysbiotic plaque and biofilm [10-13]. Henceforth, ﻿ oral pathological issues, such as caries and periodontic diseases, are recognized because of the upset equipoise between the microbiota and the human host [14,15]. Dysbiosis of the oral microbiota gives rise to a probability of pathogenic infection among internal organs through gut microbiota dysbiosis [16-18]. It arouses poor mucosal wall physiology, leading to steatohepatitis through the enterohepatic circulation [19]. Another study revealed that the oral microbiome has supervisory authority over the gut microbiome [17]. The Human Oral Microbiome Database (HOMD) makes it web accessible (www.homd.org). The HOMD includes 619 taxa in 13 phyla, as follows: *Actinobacteria, Bacteroidetes, Chlamydiae, Chloroflexi, Euryarchaeota, Firmicutes, Fusobacteria, Proteobacteria, Spirochaetes, SR1, Synergistetes, Tenericutes, and Saccharibacteria *(TM7) [20]. Furthermore, the major bacterial genera in the healthy rima oris are depicted in Table 1 [21-26].

**Table 1:** List of microbiota presence in the healthy oral cavity

Types	Cocci	Rods
Gram-positive	Abiotrophia, Peptostreptococcus, Streptococcus, Stomatococcus	Actinomyces, Bifidobacterium, Corynebacterium, Eubacterium, Lactobacillus, Propionibacterium, Pseudoramibacter, Rothia
Gram-negative	Moraxella, Neisseria, Veillonella	Bacteroidales, Campylobacter, Capnocytophaga, Desulfobacter, Dialister, Fusobacterium, Prevotella, Firmicutes, Lautropia, Desulfovibrio, Eikenella, Fusobacterium, Hemophilus, Leptotrichia, Selemonas, Simonsiella, Treponema, Wolinella

Antony van Leeuwenhoek first reported the presence of microbes' oral cavity in the late 16th century (1684) [27-29]. An American dentist and the first oral microbiologist, Willoughby Dayton Miller, earliest (1891), generated the concept that oral pathogens and infection affect other body parts, causing multiple systemic disorders [30,31]. Billings in 1913 reported that dental infection instigated several systemic diseases, for example, endocarditis, nephritis, rheumatoid arthritis, etc. [32]. A considerable portion of healthcare professionals who trust dental infection, biofilm, plaque, and their breakdown products enter the blood circulation and instigate several retrogressive, wasting, or systemic diseases. Consequently, tooth extraction became a very accepted therapeutic intervention for multiple systemic disorders [31]. The discovery of molecular procedures for microbiology, such as DNA sequencing, DNA-DNA hybridization, and polymerase chain reaction, has led to an opportunity to build a wide-ranging depiction of the assortment and symphony of the oral microbiome [29]. Our mouth orifice has the second most prevalent and assorted microbiota after the colon, embracing over 700 different microbes. The oral cavity fosters abundant microorganisms that comprise viruses, bacteria, protozoa, and fungi [28]. Dysbiosis of oral microbiota causes oral (chronic periodontitis) and multiple long-lasting inflammatory or degenerative systemic health disorders such as inflammatory bowel disease [33], squamous cell carcinoma of mouth [34,35], obesity [36], rheumatoid arthritis [37,38], type 2 diabetes mellitus (T2DM) [27,39], atherosclerosis [40], cardiovascular diseases [41], preterm birth [42], Alzheimer's disease [43], and many more [44-47].

Globally, nonalcoholic fatty liver disease (NAFLD) is a frequent instigating factor of long-lasting hepatic illness [48], with a worldwide prevalence of around 30% [49]. Schaffner and Thaler first coined the term NAFLD [50]. Ludwig et al. reported that a persistent type of fatty hepatic ailment histologically looks like alcoholic steatohepatitis, causing hepatocellular injury. Nevertheless, these liver disorder cases possess no history of alcohol consumption. This research group termed this condition nonalcoholic steatohepatitis (NASH) [51]. Two types of NAFLD have been recognized: NASH and nonalcoholic fatty liver (NAFL) [52]. NAFL is identified by the buildup of lipids in hepatic cells without causing any cellular destruction and with trivial or no inflammatory process in the hepatic lobe [53-55]. There is another hepatic disorder named metabolic dysfunction-associated fatty liver disease (MAFLD). Readers often get confused with a similar hepatic disease. MAFLD was anticipated as a more suitable name than NAFLD since this terminology preferably delineates the pathophysiology of this hepatic disorder and its related metabolic anomalies in 2020 [56,57]. MAFLD has appeared as the most frequent persistent liver disorder in both rich and poor economic nations because of the mushrooming in the frequency of overweightness, obesity, and metabolic syndrome (MetS) [58]. One meta-analysis was conducted regarding study evaluations of periodontal disease in NAFLD. "The odds ratio (1.91; 95% CI 1.21-3.02; p = 0.006) indicates that the chance of presenting periodontal disease is 91%" greater among patients with NAFLD than patients without NAFLD [59]. Chen et al. reported that affirmative relations link periodontal disorder and hepatic cirrhosis (OR = 2.28; 95% CI = 1.50-3.48) and NAFLD (OR = 1.19; 95% CI = 1.06-1.33) [60]. Aguiar et al. revealed that patients with periodontal disorders instigate the progression of NAFLD because of distorted sphingolipid metabolism that headed to intensified insulin resistance, hepatic inflammation [59], and mitochondrial dysfunction [61].

Problem statement of the study

This paper aims to investigate the complex interplay between NAFLD and periodontitis. Despite the established association between these two conditions and their shared factors, such as obesity, insulin resistance, and chronic inflammation, there remains a gap in understanding the bidirectional nature of their relationship. This study also aims to address. Questions like the extent of the influence of NAFLD on the development and progression of periodontitis. How does periodontitis contribute to the pathogenesis and exacerbation of NAFLD? What underlying mechanisms link NAFLD and periodontitis, including systemic inflammation, dysbiosis, and oxidative stress? What are the clinical implications of the bidirectional relationship between NAFLD and periodontitis regarding disease management and treatment outcomes? This research will also contribute to advancing our understanding of the systemic consequences of periodontal disease and highlight the importance of interdisciplinary collaboration between hepatologists and periodontists in optimizing patient care.

Objective of the study

The primary objective of this review is to evaluate the possible connection or association between periodontal disease and NAFLD.

## Review

Materials and methods

**Figure 1:**
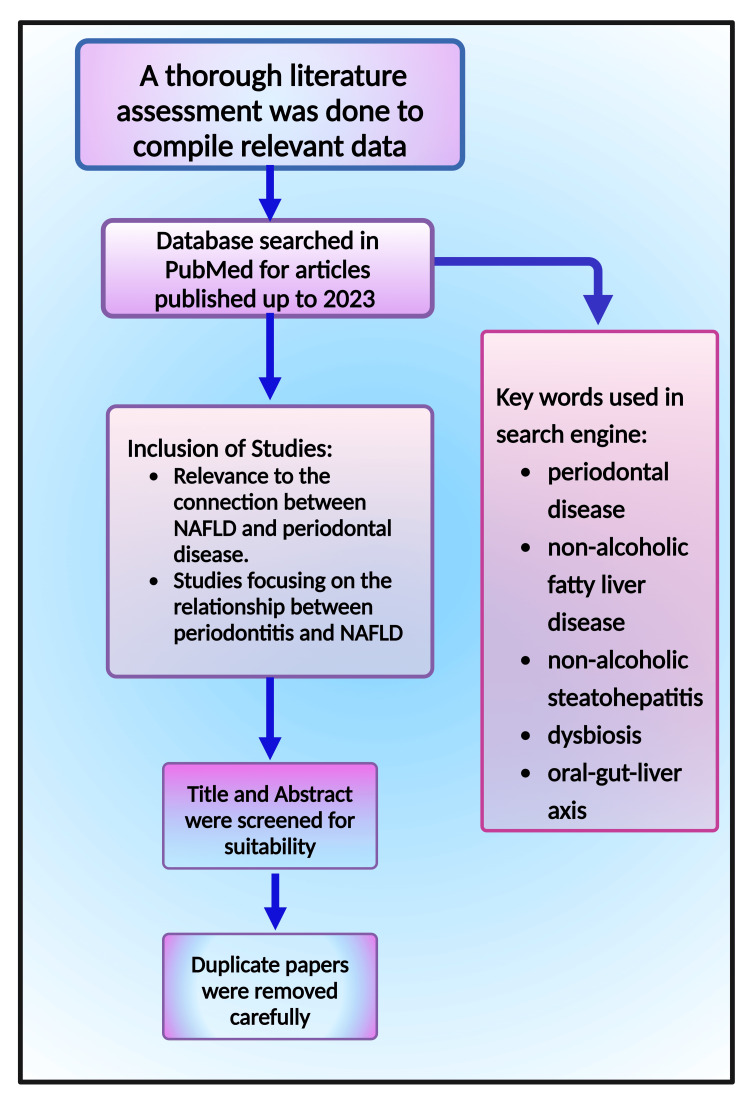
A flow chart illustrating the methodology of the study This figure was drawn using the premium version of BioRender [62], (https://biorender.com/), accessed on June 18, 2024, with the agreement license number EQ26YG04HQ Image credit: Susmita Sinha

This narrative review paper explores the prevalence and severity of periodontitis in NAFLD patients, the connection between these two disorders, and their relationship to other body systems. These were all examined through a thorough literature assessment to compile relevant data. Databases such as PubMed, Google Scholar, and Google were hand-searched for articles published up to 2023. Keywords included were "periodontal disease," AND "nonalcoholic fatty liver disease," AND "nonalcoholic steatohepatitis," AND "dysbiosis," AND "oral-gut-liver axis." The articles included were based on their relevance to the connection between NAFLD and periodontal disease. Studies focusing on the relationship between periodontitis and NAFLD were selected. The quality of the evidence, study design, and methods for evaluating the rapport regarding the association between both conditions received particular attention (Figure 1). Almost all papers were downloaded from PubMed; only one utilized the website BioRender (https://www.biorender.com/).

Review of literatures

The liver is the largest solid organ in the human body [63]. The liver engages in a dominant physiological function in metabolic homeostasis and is a paramount area for the creation, metabolism, stowage, and redistribution of carbohydrates, proteins, and lipids [64]. The liver detoxifies potentially toxic chemicals in exposed human beings [65]. The liver also processes substances like hemoglobin, bile salts, iron, vitamins, copper, and medications by absorbing, transforming, and releasing these products [66]. The liver, the largest parenchymal organ, obtains blood from the gastrointestinal tract (GIT) and systemic circulation due to its distinctive anatomical positioning. The enteric portal vein, which supplies the liver with 80% of its blood supply, is rich in nutrients, bacterial metabolites, and dietary antigens. The hepatic artery, which branches off the abdominal aorta, provides the remaining 20% and serves as the liver's nutrient vessel [67]. Hepatocytes, comprising roughly 70%-85% of the liver's volume, are the primary functional cells within the organ. They play a pivotal role in controlling a portion of its immune capabilities (innate immunity) [68,69]. Hepatocytes are the primary contributors to the creation of the liver's extracellular matrix (ECM) [70,71]. Kupffer cells represent the principal resident macrophages within the liver and the maximum number of mononuclear phagocytes in the human body, constituting the primary cell population regarding the innate immune system [72-74]. The liver possesses its regenerative ability; henceforth, it can often recover from significant damage, yet continuous or chronic damage eventually leads to various chronic liver conditions [75].

**Figure 2:**
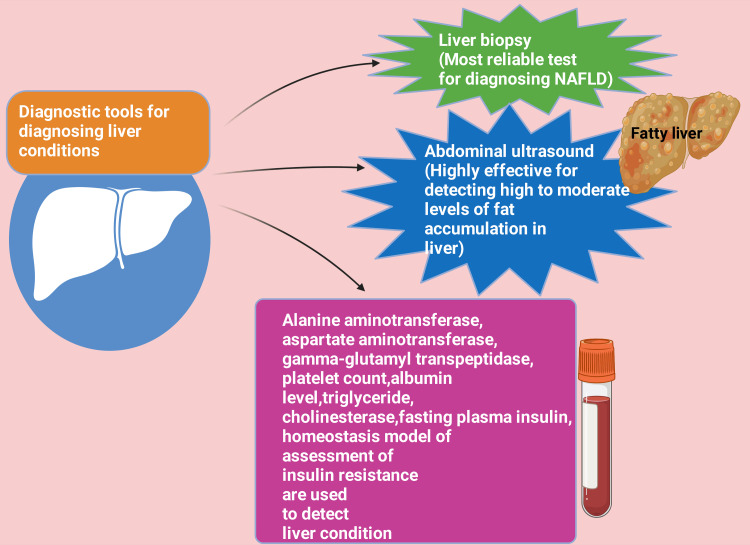
Various diagnostic tools for detecting liver conditions NAFLD: Nonalcoholic fatty liver disease This figure was drawn using the premium version of BioRender [62] (https://biorender.com/), accessed on April 27, 2024, with license number UE26QYJUJD Image credit: Rahnuma Ahmad

The most reliable method (gold standard) for diagnosing and staging NAFLD is through liver biopsy and histopathology [76]. Iwasaki et al. conducted a cross-sectional study to determine the relationship between severe periodontists and NAFLD in Japan. This research does abdominal ultrasonography to detect NAFLD. Among the research participants, around 28% had NAFLD with extreme cases of periodontal diseases. NAFLD cases had a significantly (p < 0.05) greater incidence of probing pocket depth (PPD) ≥ 4 mm than those cases who do not have NAFLD. There was a statistically significant (p < 0.01) difference observed in the adjusted odds ratios of having PPD ≥ 4 mm for NAFLD (odds ratio = 1.881; 95% CI 1.184-2.9870); after adjusting for body mass index (BMI), regular exercise habits, number of teeth present, presence of periodontitis, Brinkman index, raised serum C-reactive protein (CRP), blood pressure, sex, and age [77]. Abdominal ultrasound is highly effective at detecting both moderate and high levels of fat accumulation, making it a valuable diagnostic tool for identifying NAFLD, whether the deposits are present or absent [78,79]. Again, if distinguishing or diagnosing NAFLD from other chronic hepatic diseases becomes challenging or there's suspicion of nonalcoholic steatohepatitis, contemplating a hepatic biopsy and histopathological is the most recommended examination for appropriate diagnosis [80,81]. Indicators such as serum alanine aminotransferase (ALT), aspartate aminotransferase (AST), gamma-glutamyl transpeptidase, platelet count, albumin levels, triglycerides, cholinesterase, fasting plasma insulin, homeostasis model of assessment of IR, and various other biomarkers have been utilized to assess liver conditions (Figure 2) [82-84]. The blood indicators GGT, ALT, and AST are measured in liver function tests [85]. Key enzymes in the metabolism of amino acids, AST and ALT, are found in the cells of essential organs suchas the liver, kidneys, and heart [86-88].

Common risk factors for NAFLD

**Figure 3:**
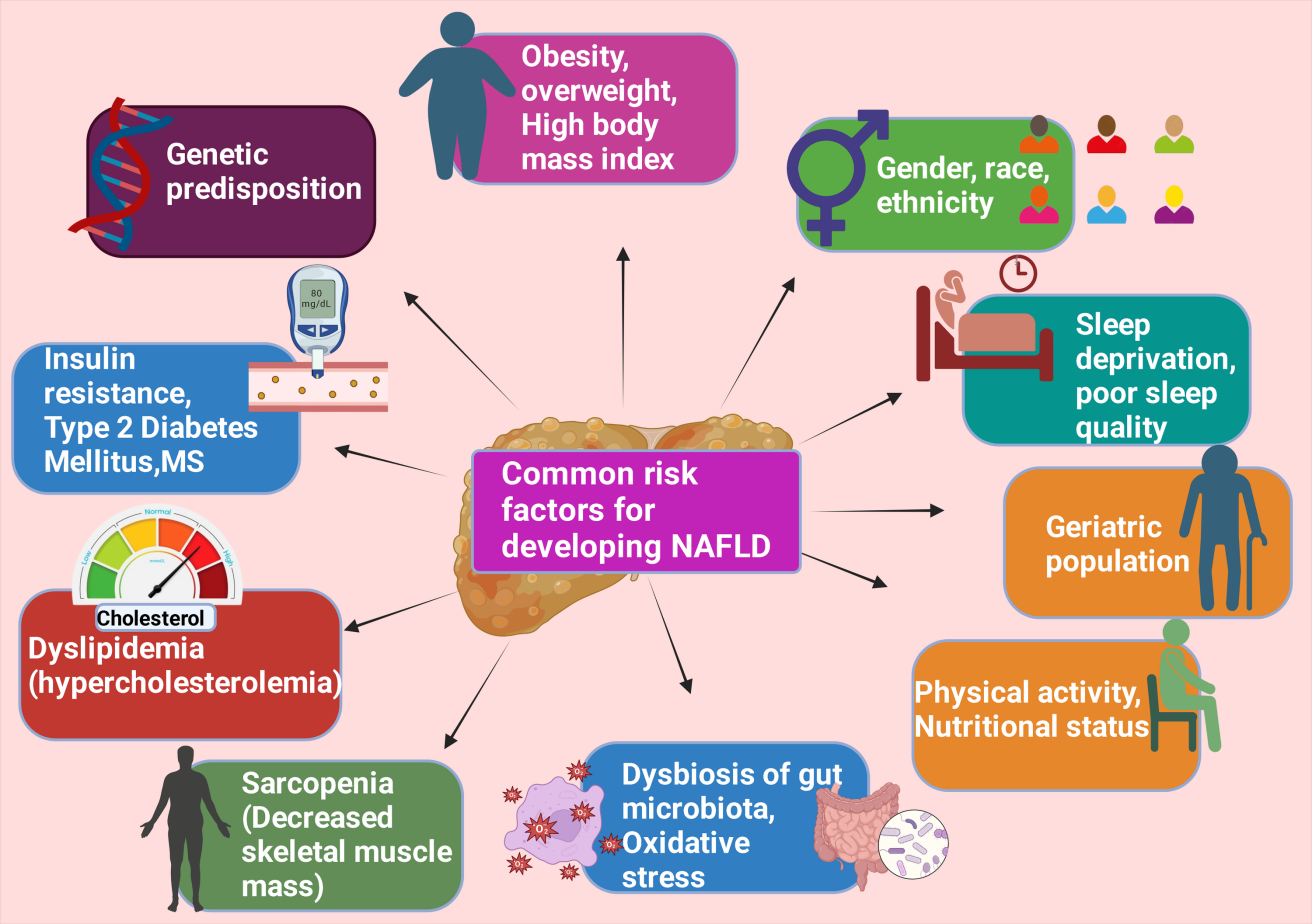
Illustrates the common risk factors for developing NAFLD NAFLD: Nonalcoholic fatty liver disease; MS: metabolic syndrome; O_2_: super oxide This figure was drawn using the premium version of BioRender [62] (https://biorender.com/), accessed on June 16, 2024, with the agreement license number GS26Y3XTVM Image credit: Rahnuma Ahmad

Genetic tendencies, gender, geriatric community, race, ethnicity, poor sleep quality and sleep deprivation, physical activity, nutritional status, dysbiosis of the gut microbiota, increased oxidative stress, overweight, obesity, higher BMI, insulin resistance (IR), T2DM, MetS, dyslipidemia (hypercholesterolemia), and sarcopenia (decreased skeletal muscle mass) are common risk factors for developing NAFLD (Figure 3) [89,90].

Oral-gut-liver axis

"The oral-gut-liver axis is a concept that aims to understand the potential relationships between different organs, particularly the oral cavity, gut, and liver" [91]. Individuals suffering from periodontitis possess a distinctive dysbiotic oral microbiome [92], leading to the continuous, unwitting ingestion of pathogens and noxious metabolites from the saliva and dental plaque to the gut through blood circulation or enteral paths [93,94]. Dysbiotic oral pathogens, when translocating to the gastrointestinal tract, similarly cause gut dysbiosis [95]. These pathogenic microbes and lethal metabolites enter the liver through the portal vein, causing adverse hepatic consequences [96]. These metabolites are derived from dysbiotic oral pathogens, often known as pathogen- and damage-associated molecular patterns (PAMPs; DAMPs) [92]. They activate a predisposed host immune system in the periodontal disease setting, which could influence the escalation of a range of systemic pro-inflammatory cytokines and chemokines [97,98], increasing the possibility of impairing the hepatic physiology function [92,99]. It has been reported that the gut microbiota is reflected as a biological barricade, defending against settling and expanding the population of pathogenic microbes within the GIT [100]. An impaired gut microbiota disrupts the integrity of intestinal tight junctions, leading to heightened mucosal permeability [82,100,101]. Analogously, hepatic poor performance function also worsens the existing disease process and clinical sequels within the buccal cavity [102].

NAFLD and periodontitis

Kobayashi et al. reported that the way of living associated with various severe illnesses such as ﻿ hepatic disorders, NASH, and NAFLD also has a potential connection with periodontal diseases [103]. The manner of living remains the independent risk factor that has the potential to be alterable for several noncommunicable diseases (NCDs) [104-106]. The pathogenesis of NAFLD remains not definitively known, although this disease is quite common around the globe [107]. Nevertheless, several precipitating factors are common for developing ﻿periodontitis and NAFLD [108]. Furthermore, the progression of both these diseases leads to the excess synthesis of systemic pro-inflammatory signaling proteins and ﻿reactive oxygen species (ROS); IR intensifies the process and is mainly observed among T2DM and obese cases [108-111]. Kuroe et al. ﻿reported that those cases of moderate to severe periodontitis augmented the probability of emerging liver fibrosis with an odds ratio of 2.06 (0.89-4.76) among the Japanese population [112]. Additionally, NAFLD-related advanced fibrosis usually observes reduced microbial multiplicity, frequently complemented by the high level of Gram-negative microbes [113,114]. Helenius-Hietala et al. revealed that advanced cases of severe periodontitis when concurrently diagnosed with NAFLD among the Finnish population require immediate hospitalization with fatal clinical outcomes and hepatic carcinoma [115].

Red complex pathogenic microbes most frequently instigate periodontal disease, including *P. gingivalis*, *Treponema denticola*, and *Tannerella forsythia *(*Bacteroides forsythias*) [116,117]. *Porphyromonas gingivalis* (a Gram-negative anaerobic bacteria) is one of the principal pathogenic microbes extensively found among patients with periodontal disorders [107,118]. *P gingivalis* infection has been revealed to be responsible for the deterring feature for sprouting IR, NAFLD, NASH, and MetS [107,119]. Elevated *P. gingivalis *levels in the oral cavity, elevated endotoxin levels, and elevated antibody titers for *P. gingivalis *are linked to amplified hepatic rigorousness, which can be detected by MRE [120,121]. Periodontopathic microbes such as *P. gingivalis* flow down from the mouth to the gut and directly enter the blood through a bleeding gum, a typical feature of periodontitis [122,123]. Periodontopathic *P. gingivalis* later instigates gut dysbiosis [124]. *P. gingivalis* provokes intestinal microenvironmental disorder and increases the permeability of the intestinal wall, thus facilitating the direct passage of pathogenic microbes and their metabolites to the bloodstream and hepatic tissues [125-127]. Thus, periodontal pathogenic microbes and their metabolites in hepatic tissue instigate hepatic inflammation and the progression of fatty liver disease [19,82,103,128].

**Figure 4:**
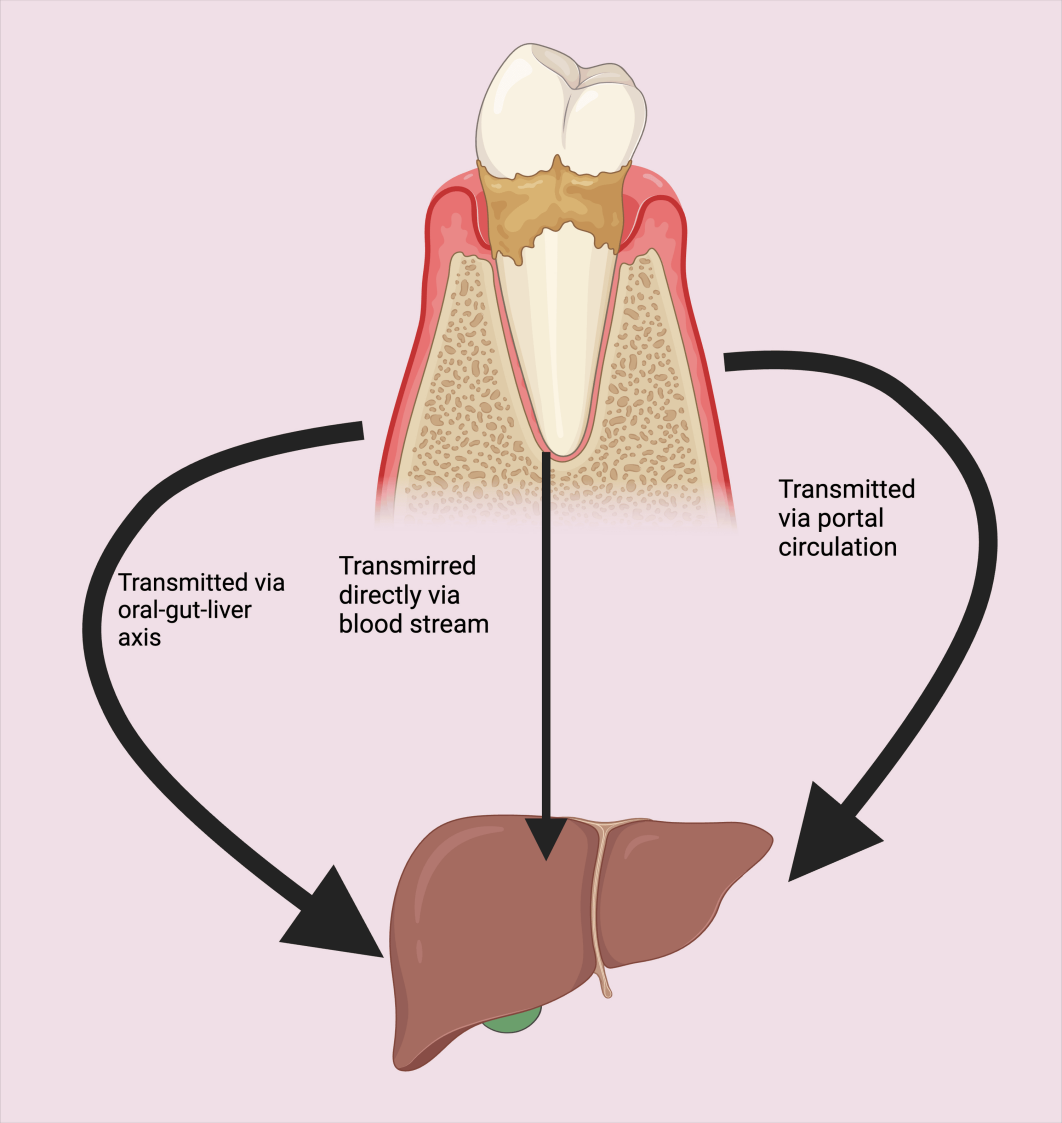
Pathways showing the connection of oral microbiota with the liver This figure was drawn using the premium version of BioRender [62] (https://biorender.com/), accessed on June 20, 2024, with the agreement license number UY26YPY3V Image credit: Hardika Vegda

Periodontal-disease-producing pathogens can synthesize inflammatory mediators such as tumor necrosis factor-alpha (TNF-α), interleukin 6 (IL-6), and interleukin-1 family (IL-1), which results in inflammation-triggering vascular endothelial damage and when entering the blood instigates systemic inflammation [44,129]. Periodontal pathogens trigger neutrophils and monocytes, producing excessive pro-inflammatory cytokines [129]. Hence, systemic inflammations are frequently linked with periodontitis and the development of IR [129,130] by increasing the levels of serum adipocytokines (TNF-α, IL-6, and IL-1) [44,129]. Additionally, these Gram-negative anaerobic periodontal pathogens produce lipopolysaccharides (LPS). LPS “is a virulence factor of Gram‐negative bacteria with a crucial importance to the bacterial surface integrity” [131]. Furthermore, circulating LPS can potentiate ongoing inflammation and oxidative stress [132]. LPS binds activated Toll-like receptors (TLRs) such as TLR-4, which increases oxidative stress and chronic inflammation [108,133,134]. Therefore, all these pathogenic difficulties further potentiate additional harm to the liver's parenchymal tissue (Figure 4).

It has been determined that oral and intestinal dysbiosis are considerable risk factors for chronic liver disorders. Subsequently, the inflammatory mediators promote adverse impacts on the liver and bring forward an essential clinical and public health issue [91]. The earliest medical and surgical intervention of chronic periodontal diseases can potentially decrease endotoxin levels and enhance the clinical outcome of NAFLD [44].

Patients with NAFLD had considerably higher rates of *P. gingivalis* in their saliva (detected through polymerase chain reaction (PCR)) and hepatic tissue (determined through biopsy and histopathology) than in non-NAFLD healthy subjects [103,135,136]. Multiple studies similarly reported that a higher number of *P. gingivalis* consider ﻿magnetic resonance elastography (MRE)-appraised hepatic tautness [41,120,136,137]. MRE-determined hepatic stiffness was observed more among NAFLD patients with higher levels of *P. gingivalis* FDC381 and SU63 antibody levels [120,138]. Multiple research projects reported that periodontal pockets of 10 or more and a depth of 4 mm or more are considerably associated with hepatic stiffness [103,120]. 

However, *P. gingivalis* is not the solitary contributor to NAFLD pathogenesis [139]. ﻿*Selenomonas noxia* and *Streptococcus oralis* serum antibodies possess a substantial relationship with the development of severe periodontal fatty liver disease [82,139]. ﻿*Tannerella forsythia* and *Treponema denticola *antibody titers possess a strong relationship in causing severe periodontitis, nevertheless having a weak association with the advancement of hepatic ﻿steatosis [139]. *T. denticola *was notably more frequently detected in NAFLD patients compared to the frequency observed in control subjects [135].

**Figure 5:**
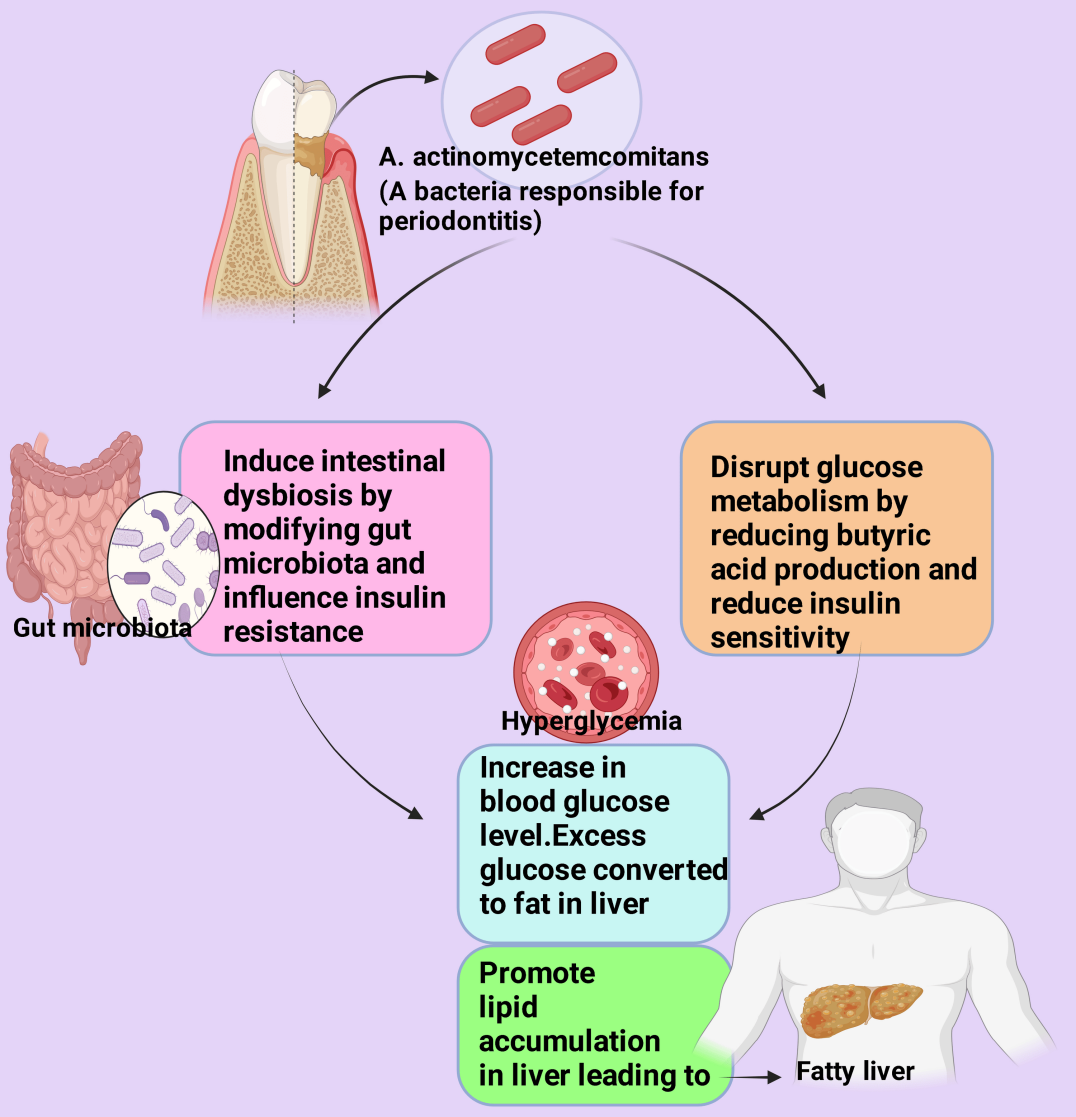
The mechanism by which A. actinomycetemcomitans causes fatty liver This figure was drawn using the premium version of BioRender [62] (https://biorender.com/), accessed on April 27, 2024, with the agreement license number MA26R10OCD Image credit: Rahnuma Ahmad

*Aggregatibacter actinomycetemcomitans *is often called “a tooth killer” [140]. *A. actinomycetemcomitans *is another pathogen that can persuade gastrointestinal microbiome commotion and disrupt glucose metabolism [141,142]. This pathogen synthesizes both endotoxin and exotoxin to trigger an inflammatory response ﻿by collaborating with the TLR4 [143-145]. Various research demonstrates that a high-fat diet is linked to dysbiosis in the gastrointestinal microbiome of humans [146,147]. Genus *Turicibacter *of *Firmicutes *phylum is a usual microbiota component of the human gastrointestinal tract as normal flora [148,149]. It is involved in host lipids, bile acids, and steroid metabolism [150,151]. Its richness is inverse as microbiota in the human intestine are strongly associated with inflammatory bowel diseases, e.g., Crohn’s disease [151] and tryptophane/serotonin metabolism [152]. *Turicibacter* has been reported to increase butyric acid synthesis (a short-chain fatty acid) in human cases [153,154]. An increase in butyric acid is associated with improved insulin secretion and sensitivity and possesses substantial anti-obesity and anti-inflammatory effects [154-157]. It has been reported that oral periodontal pathogen *A. actinomycetemcomitans *causes dysbiosis of the gut microbiota that includes the genus *Turicibacter* [141,158,159]. *A. actinomycetemcomitans* may modify the gut microbiota to influence IR but reduce butyric acid production (Figure 5). Multiple studies have examined the impact of treating *A. actinomycetemcomitans* with antibiotics on the gut microbiota and glucose metabolism, resulting in enhanced glucose tolerance, reduced liver lipid accumulation, and alterations in the gut microbiota [160-163].

Patients with periodontitis exhibited elevated CRP levels [164], whereas those with gingivitis demonstrated elevated gamma-glutamyl transferase (GGT) levels [83]. GGT is a hepatic plasma-membrane-bound enzyme primarily produced in liver cells. It is also synthesized or found on the plasma membranes of almost all vital organ epithelial tissues with secretory or absorbing functions and cells [165-168]. The principal physiological function of GGT is the extracellular synthesis of the thiol antioxidant, glutathione [169]. Glutathione shields cells antagonistic toward oxidants constructed during regular metabolic processes [170]. GGT, therefore, plays an essential role in cellular defense. Whitfield [164] reported that GGT is a conventional biomarker of hepatic diseases, bile duct disorders, alcohol drinking-related hepatic illness, T2DM, coronary heart disease, and stroke [171]. Additionally, raised GGT was also observed in heart failure, hyperthyroidism, MetS, and pancreatitis [172-175].

Alcohol consumption is known to reduce GGT production [171,176]. Explicitly, for every one-unit escalation in “GGT/high-density lipoprotein cholesterol (HDL-C) ratio, the prevalence of NAFLD will increase by 0.3%. As GGT/HDL-C ratio quartiles increased, the prevalence of NAFLD/MetS in Q4 (highest GGT/HDL-C ratio quartile) was 6.362/3.968 times higher than that in Q1 (lowest GGT/HDL-C ratio quartile)” [177]. Individuals with chronic periodontitis exhibit elevated protein carbonyl (PC) levels in serum and the gingival crevicular fluid (GCF) [178]. Chronic severe periodontitis could potentially result in heightened inflammatory biomarkers including GGT levels in the GCF [179,180]. Furthermore, high periodontal inflamed surface area (PISA) had raised GGT levels. PISA is currently considered a new marker for chronic destructive periodontal disease, hepatic disorders, and related systemic illness [83,181,182]. It has been reported that therapeutic intervention, e.g., scaling and root planning (SRP), considerably cut down hepatic enzyme (AST, ALT) and endotoxin among cases of chronic destructive periodontal disease and NAFLD [103,138,183,184]. SRP's primary goals are removing biofilm calculus and “to create a biologically compatible root surface and reduce the inflammatory burden” [185]. SRP treatment suggestively minimized liver fat content (LFC). Endotoxin and ﻿ liver enzyme levels in patients with NAFLD and periodontal disease were mainly well-accepted in these patients [138]. One pharmacological agent, lubiprostone, a laxative, minimizes hepatic enzymes in patients with NAFLD and constipation by blocking endotoxin leakage [186,187]. Lubiprostone (the ClC-2 chloride channel activator) is well-accepted among patients. It reduces intestinal penetrability by synthesizing additional colonic mucus through its prostaglandin-like properties on the intestine and improves repairing tight junction proteins (TJPs) of the intestine [187,188]. Lubiprostone alters lipid metabolism and improves NAFLD [187].

Multiple Japanese studies revealed a substantial association between nonalcohol drinkers, the presence of probing pocket depths of 4 mm or greater, and higher serum ALT levels [77,120,189]. Ahmad et al. also demonstrated that the coexistence of MetS and elevated serum ALT was positively associated with pocket depth in adult males with minimal alcohol consumption [190].

**Figure 6:**
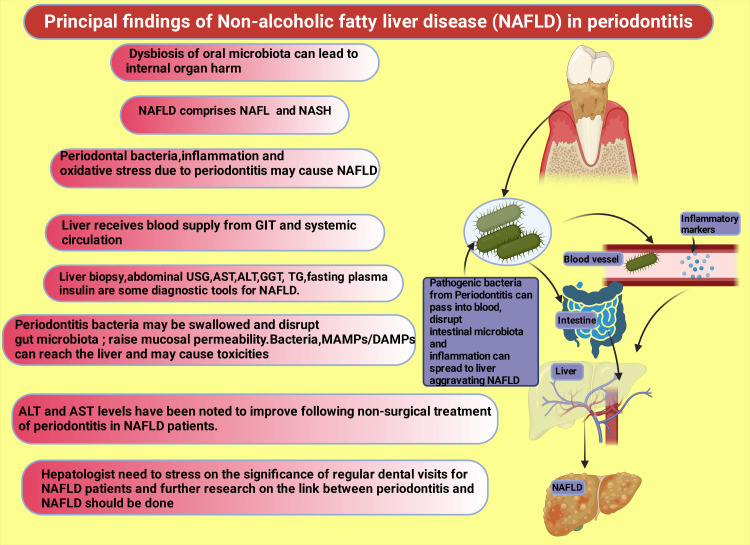
Principal findings of nonalcoholic fatty liver disease in periodontitis ALT: Alanine aminotransferase; AST: aspartate aminotransferase; GGT: gamma-glutamyl transferase; MAMP: microbe-associated molecular patterns; DAMPs: damage-associated molecular pattern; *P. gingivalis*: *Porphyromonas gingivalis*; *T. denticola*: *Treponema denticola*; *T. forsythia*: *Tannerella forsythia*; *A. actinomycetemcomitans*:* Aggregatibacter actinomycetemcomitans* This figure was drawn using the premium version of BioRender [62] (https://biorender.com/), accessed on June 22, 2024, with the agreement license number AS26Z02LUJ Image credit: Rahnuma Ahmad

Multiple research studies revealed that timely medical and surgical intervention regarding chronic periodontal diseases improves serum lipid profiles toward positive, controls blood glucose, increases insulin sensitivity, decreases IR, decreases inflammatory markers, including endotoxin, and restores overall microbiota [128,138,191-194]. Thus, chronic periodontitis and NAFLD cases are improved. Figure 6 reveals the principal findings of this narrative review.

Limitations of this paper

This review's drawback was its exclusive dependence on the previously published documents from explorations of “computerized databases, hand searches, and authoritative texts” [195]. This is a narrative review; therefore, no patient data was collected. Articles only written in English were included. No attempt was made to conduct a systematic review and meta-analysis. Narrative reviews have built-in confines in footings of neutrality, comprehensiveness of literature exploration, and clarification of findings [195-199]. ﻿Narrative reviews cannot comprise based on science for dedicated issues, nor do they propose conclusive assertions to develop guidelines [196]. In opposing earlier findings, Greenhalgh et al. reported that “narrative reviews provide interpretation and critique; their key contribution is deepening understanding” [200]. Henceforth, the narrative review is considered a different way of disseminating science, knowledge, wisdom, sagacity, and many other issues; nevertheless, it is not an unfortunate colleague of the systematic review [201,202].

Future recommendations regarding management of NAFLD

Extensive research is needed to elucidate the pathophysiological mechanisms linking NAFLD and periodontitis. Understanding these connections on a molecular and systemic level is crucial for developing targeted interventions. We should focus on creating effective pharmacological treatments that can interrupt the various pathways through which NAFLD and periodontitis interact. This includes identifying specific biomarkers and therapeutic targets. Healthcare professionals must spearhead policy and public health initiatives. We need to develop and enforce policies that educate healthcare professionals and the public about the profound impact of poor oral health on overall health, particularly its role in exacerbating conditions like NAFLD.

Public awareness campaigns should be launched to raise awareness about the importance of oral hygiene. These campaigns should emphasize the connection between oral health and systemic conditions, including liver diseases like NAFLD. Integrate oral health education into existing health promotion programs. Educate the public on effective oral hygiene practices, the significance of regular dental check-ups, and the broader health implications of maintaining good oral health.

Encourage a collaborative approach between dental and medical healthcare providers to manage and prevent NAFLD and related conditions. This includes regular screenings for periodontal disease in patients with NAFLD and vice versa. Implement routine oral health assessments as part of the standard care protocol for patients at risk of or diagnosed with NAFLD. Early detection and management of periodontal disease can mitigate its impact on liver health. By advancing research, promoting public awareness, and integrating healthcare services, we can develop a more comprehensive strategy to manage and potentially reduce the incidence of NAFLD and its associated complications.

## Conclusions

Growing evidence from scholastic scientific works exhibits that there is a correlation between NAFLD and periodontitis. There was a noteworthy and statistically significant drop in blood albumin levels when comparing NASH/NAFLD individuals who tested positive for *P. gingivalis* to those who did not. These findings suggest that a decline in liver function could progress more rapidly in patients positive for *P. gingivalis*. Considering these findings, chronic inflammation, IR associated with obesity, and changes brought on by periodontal disease in the gut microbiota might contribute to the development of NAFLD. Other risk factors for NAFLD development include genetic tendencies, gender, geriatric population, race, ethnicity, metabolic syndrome, oxidative stress, physical activity, sleep quality, and nutritional status. Therefore, this study concludes that hepatologists should remind patients with NAFLD of the significance of routine dental visits. As new findings suggest a potential connection between NAFLD and periodontitis, forthcoming research is crucial to ascertain the strength of these links and understand the biological pathways that interconnect these conditions. Additionally, there's a possibility of a two-way relationship where NAFLD could impact periodontitis outcomes, necessitating further exploration.
